# Histone Deacetylase SIRT1 Mediates C5b-9-Induced Cell Cycle in Oligodendrocytes

**DOI:** 10.3389/fimmu.2020.00619

**Published:** 2020-04-09

**Authors:** Alexandru Tatomir, Gautam Rao, Dallas Boodhoo, Sonia I. Vlaicu, Austin Beltrand, Freidrich Anselmo, Violeta Rus, Horea Rus

**Affiliations:** ^1^Department of Neurology, University of Maryland School of Medicine, Baltimore, MD, United States; ^2^Department of Neurosciences, “Iuliu Hatieganu” University of Medicine and Pharmacy, Cluj-Napoca, Romania; ^3^Department of Internal Medicine, “Iuliu Hatieganu” University of Medicine and Pharmacy, Cluj-Napoca, Romania; ^4^Division of Rheumatology and Immunology, Department of Medicine, University of Maryland School of Medicine, Baltimore, MD, United States; ^5^Research Service, Veterans Administration Maryland Health Care System, Baltimore, MD, United States

**Keywords:** C5b-9, oligodendrocyte, SIRT1, cell cycle, cyclin D1

## Abstract

Sublytic levels of C5b-9 increase the survival of oligodendrocytes (OLGs) and induce the cell cycle. We have previously observed that SIRT1 co-localizes with surviving OLGs in multiple sclerosis (MS) plaques, but it is not yet known whether SIRT1 is involved in OLGs survival after exposure to sublytic C5b-9. We have now investigated the role of SIRT1 in OLGs differentiation and the effect of sublytic levels of C5b-9 on SIRT1 and phosphorylated-SIRT1 (Ser27) expression. We also examined the downstream effects of SIRT1 by measuring histone H3 lysine 9 trimethylation (H3K9me3) and the expression of cyclin D1 as a marker of cell cycle activation. OLG progenitor cells (OPCs) purified from the brain of rat pups were differentiated *in vitro* and treated with sublytic C5b-9 or C5b6. To investigate the signaling pathway activated by C5b-9 and required for SIRT1 expression, we pretreated OLGs with a c-jun antisense oligonucleotide, a phosphoinositide 3-kinase (PI3K) inhibitor (LY294002), and a protein kinase C (PKC) inhibitor (H7). Our data show a significant reduction in phospho-SIRT1 and SIRT1 expression during OPCs differentiation, associated with a decrease in H3K9me3 and a peak of cyclin D1 expression in the first 24 h. Stimulation of OLGs with sublytic C5b-9 resulted in an increase in the expression of SIRT1 and phospho-SIRT1, H3K9me3, cyclin D1 and decreased expression of myelin-specific genes. C5b-9-stimulated SIRT1 expression was significantly reduced after pretreatment with c-jun antisense oligonucleotide, H7 or LY294002. Inhibition of SIRT1 with sirtinol also abolished C5b-9-induced DNA synthesis. Taken together, these data show that induction of SIRT1 expression by C5b-9 is required for cell cycle activation and is mediated through multiple signaling pathways.

## Introduction

Oligodendrocytes (OLGs) are the myelin-forming cells of the CNS. They form the myelin sheath by wrapping their plasma membrane around axons. OLGs are derived from progenitor cells called OPCs, which migrate into gray and white matter and differentiate into mature OLGs. This process is highly active during CNS development and after birth, but a subpopulation of OPCs persists in the adult brain, where these cells contribute to OLGs replacement and remyelination during normal states, such as aging, or during pathological conditions (1, 2).

Oligodendrocytes and myelin are the main targets of autoimmune attack in MS, a chronic, demyelinating disease of the CNS. OLGs are particularly susceptible to complement attack, since they lack some of the key membrane regulatory molecules that normally inhibit complement assembly (3, 4). Terminal complement C5b-9 complex [also called membrane attack complex (MAC)] can assemble on OLGs membranes and promote cell death at lytic concentrations (5). However, when newly differentiated OLGs are exposed to sublytic doses of C5b-9, these cells can instead enter the S phase of the cell cycle, by activating cyclin-dependent kinases important for G1 phase progression and increasing the expression of the proto-oncogenes c-jun, c-fos, and junD (6). In addition to cell cycle activation, there is also a downregulation of the expression of myelin-specific proteins, MBP and PLP (6, 7). These molecular changes indicate that sublytic C5b-9 induces a progenitor-like phenotype in OLGs that may be critical for their survival in an inflammatory environment and important for regaining myelinating potential once the inflammation subsides (6). Interestingly, studies have shown that sublytic C5b-9 can induce activation of multiple signal transduction pathways, including Ca^2+^ influx, PKC activation (7–9) and phosphoinositide-3-kinase (PI3K) / Akt-mediated inhibition of apoptosis, by inhibiting caspase 8 activation and the cleavage of the pro-apoptotic factor Bid and downregulating Fas ligand expression (10–12). In addition, sublytic C5b-9 increases the expression of the voltage-gated potassium channel K(v)1.3, which controls C5b-9-induced Akt activation, providing another mechanism through which sublytic C5b-9 induces the cell cycle in OLGs (13).

SIRT1 is a NAD-dependent class III histone deacetylase (HDAC) involved in the regulation of transcription, apoptosis, metabolism, and differentiation. It catalyzes the removal of acetyl groups from a variety of protein substrates and promotes histone H3 lysine 9 (H3K9) methylation, contributing to heterochromatin formation and transcription silencing (14, 15). We have found that SIRT1 is co-localized with surviving OLGs in MS plaques and is also expressed by astrocytes and CD4- and CD68-positive cells in MS brains (16). We have also shown that SIRT1 expression is reduced in peripheral blood mononuclear cells of MS patients during relapses and in those who do not respond to treatment with glatiramer acetate, a disease-modifying drug used for treating MS, suggesting that SIRT1 could serve as a biomarker of disease activity and patient responsiveness to therapy (15, 17). Other studies have indicated that SIRT1 is involved in OPCs proliferation and differentiation toward OLGs during pathological conditions (18–20). However, it is not currently known whether SIRT1 is implicated in the OLG cell cycle activation induced by sublytic C5b-9 or whether complement activation and sublytic C5b-9 have any effect on SIRT1.

We therefore investigated the expression of SIRT1 and p-SIRT1 during differentiation and after exposure to sublytic C5b-9. Our data indicate that sublytic C5b-9 induces cell cycle activation by increasing SIRT1 levels in a c-jun, PI3K- and PKC-dependent manner. C5b-9-mediated MBP and PLP down-regulation may also play an important role in inflammatory demyelination by affecting the ability of OLGs to express myelin proteins. This phenotype, which resembles those of precursor cells, may be helpful in mediating OLGs survival in an inflammatory environment.

## Materials and Methods

### Cultures of Primary Rat OLGs

Oligodendrocytes progenitors were isolated from neonatal Sprague-Dawley rat brains as previously described (6). After the removal of the meninges, brains were minced and passed through nylon meshes, and the mixed glial cells were cultured for 10 days in DMEM/Ham’s F-12 medium supplemented with 10% fetal bovine serum. OLG progenitor cells were isolated from the adherent astrocytes by shaking overnight at 200 rpm on a rotary shaker, then cultured in 25 cm^2^ flasks pre-coated with poly-L-lysine in an OLG differentiation medium that contained DMEM/Ham’s F-12 supplemented with transferrin (500 ng/ml), insulin (75 ng/ml), basic fibroblast growth factor (75 μg/ml), and 1mM sodium pyruvate for up to 72 h. The cells were incubated at 37°C in a 95% air / 5% CO_2_ incubator. On average, 90% of the cells were identified as OLGs, as judged by galactocerebroside (GC) staining (21).

### Activation of Serum Complement and Sublytic C5b-9 Assembly

Normal human serum (NHS) from healthy adult donors was used as a source of serum complement. The hemolytic activity of complement was inactivated by heating NHS at 56°C for 45 min (HI-NHS); this treated HI-NHS was then used as a control. OLGs were incubated with a dilution of NHS confirmed to be sublytic (7) or with HI-NHS for different periods of time. In addition, we assembled C5b-9 from purified complement components (Complement Technology, Inc., Tyler, TX, United States; Quidel, San Diego, CA, United States) as previously described (10, 11, 22). In brief, OLGs were incubated at 37°C with 18 U of C5b6 and 10 μg/ml each of C7-C9 in a final volume of 2 ml for the indicated periods of time. Inactive C5b6 (18 U) was used as a control. The concentrations of complement proteins used in this study were sublytic for OLGs, as determined by staining cells with the vital dye trypan blue and by measuring release of cytoplasmic lactate dehydrogenase as an indicator of cell death (11, 22).

### Signal Transduction Pathways Inhibition

Oligodendrocytes were differentiated *in vitro* for 72 h and then pretreated with a c-jun antisense oligonucleotide (20 μM) prepared as described (6), with the PKC inhibitor H7 at 60 μM (Bio-Techne, Minneapolis, MN, United States), or with the PI3K inhibitor LY294002 at 10 μM (Cell Signaling Technology, Danvers, MA, United States). Cells were then stimulated with sublytic C5b-9 for the indicated periods of time.

### RNA Isolation, cDNA Synthesis, and Quantitative Real-Time PCR

Total RNA obtained was purified using the RNeasy Mini Kit (Qiagen, Germantown, MD, United States) according to the manufacturer’s instructions. RNA (0.5 μg per sample) was mixed with RT buffer, dNTP, and oligo-dT primer (Invitrogen, Carlsbad, CA, United States). The RNA was denatured by incubation at 65°C for 5 min. Reverse transcriptase (Promega, Madison, WI, United States) and RNase inhibitor (Invitrogen) were then added, and the reaction mixture was incubated at 37°C for 1 h. The reaction was terminated by incubation of the mixture at 95°C for 5 min (16). Forward and reverse primers for SIRT1, MBP, SOX10, NG2/CSPG4 and PLP were synthetized by Integrated Device Technology (Coralville, IA, United States). 18S RNA (Integrated Device Technology) was used as an endogenous control. Real time PCR primers sequences are listed in Table 1. Real-time PCR was performed according to the manufacturer’s protocol using a FastStart Universal SYBR Green Master (Roche, Indianapolis, IN, United States) and StepOnePlus Real Time PCR System (Applied Biosystems, Foster City, CA, United States). Quantification was performed by using the ΔΔCT method of relative quantification as previously described (23).

**TABLE 1:** Primers used for real time PCR.

**Gene symbol**	**Primers sequence**
CSPG4	For: 5′- GGTCACATCTCCACCACATT -3′
	Rev: 5′- GTACCATGAGCAGGACGTTAG -3′
MBP	For: 5′- ACACAAGAACTACCCACTACGG -3′
	Rev: 5′- GGGTGTACGAGGTGTCACAA -3′
PLP	For: 5′- CCACCTGTTTATTGCTGCAT -3′
	Rev: 5′- TGTGGTTAGAGCCTCGCTATT -3′
SIRT1	For: 5′- CGCTTTCCCATTCGGTTTCC -3′
	Rev: 5′- TATGGACCTATCCGTGGCCT -3′
SOX10	For: 5′- GACACTCAATGGACACTCTGAT -3′
	Rev: 5′- AGGTGAGGGATGAGGTTACT -3′
18S	For: 5′- GTAACCCGTTGAACCCCATT -3′
	Rev: 5′- CCATCCAATCGGTAGTAGCG -3′

### Western Blotting

Western blotting was performed as previously described (17). Cells were lysed in RIPA buffer containing protease and phosphatase inhibitors. Whole-cell lysates (total protein=10–30 μg) were electrophoresed on 10% SDS-PAGE, followed by transfer to nitrocellulose membranes. The primary antibodies used are listed in Table 2. Membranes were first incubated with rabbit IgG against phospho-SIRT1 (Biorbyt, Cambridge, United Kingdom) overnight at 4°C then with anti-rabbit HRP-conjugated IgG (Santa Cruz Biotechnology, Dallas, TX, United States) for 1 h at room temperature, and immune complexes were visualized by using enhanced chemiluminescence (ECL) (Denville Scientific Inc., Metuchen, NJ, United States) and autoradiography. After ECL visualization, we stripped the membrane with Restore Western Blot Stripping Buffer (Thermo Fisher Scientific, Waltham, MA, United States) and reprobed the membrane for SIRT1 (Cell Signaling Technology, Danvers, MA, United States), H3K9me3 (Active Motif, Carlsbad, CA, United States), cyclin D1 (Cell Signaling Technology), and MBP (GenScript, Piscataway, NJ, United States), following similar steps as described for phospho-SIRT1. Mouse monoclonal IgG anti-β-actin and mouse monoclonal IgG anti-β-tubulin (Proteintech, Rosemont, IL, United States) were used as housekeeping controls. Anti-rabbit or anti-mouse HRP-conjugated antibodies (Santa Cruz Biotechnology) were used as secondary antibodies with 1-h incubation at room temperature. The radiographic band density was measured using UN-SCAN-IT software, version 7.1 (Silk Scientific, Orem, UT, United States).

**TABLE 2:** Antibodies used for western blotting.

Primary antibodies					
Antigen	Species	Clone	Dilution	Source	Catalog no.
Phospho-SIRT1 (Ser27)	Rabbit	Polyclonal	1:400	Biorbyt, Cambridge, United Kingdom	orb6958
SIRT1	Rabbit	D1D7	1:1000	Cell Signaling Technology, Danvers, MA, United States	9475
Cyclin D1	Rabbit	DCS6	1:1000	Cell Signaling Technology	2926
H3K9me3	Mouse	2AG-6F12-H4	1:5000	Active Motif, Carlsbad, CA, United States	39286
MBP	Rabbit	Polyclonal	1:1000	GenScript, Piscataway, NJ, United States	A01407
β-actin	Mouse	2D4H5	1:35000	Proteintech, Rosemont, IL, United States	66009-1-Ig
β-tubulin	Mouse	1D4A4	1:30000	Proteintech	66240-1-Ig

### [^3^H] Thymidine Incorporation

DNA synthesis was measured by [^3^H] thymidine incorporation assays, as previously described (6). To determine the role of c-jun in cell cycle activation induced by C5b-9, OLGs were incubated with 20 μM sense (5′-GGTCGTTTCCATCTTTGC-3′) or antisense (5′-GCAAAGAT GGAAACGACC-3′) c-jun oligonucleotides at 37°C for 4 h before exposure to activated complement. To determine the role of SIRT1 in cell cycle activation OLGs were preincubated with Sirtinol (200 μM) before exposure to sublytic C5b-9. In brief, OPCs were differentiated for 72 h, then pretreated with c-jun antisense (20 μM), c-jun sense, or sirtinol (200 μM) and then incubated with sublytic C5b-9 in the presence of 1 μCi/ml of [^3^H] thymidine (PerkinElmer, Waltham, MA, United States). OLGs were then incubated at 37°C with 5% CO_2_ for 18 h, followed by lysis and precipitation of DNA via the addition of 20% trichloroacetic acid. The precipitated DNA was filtered through Whatman GF/A filter paper (Sigma, St. Louis, MI, United States), and the filter paper was air-dried. Radioactivity was counted by liquid scintillation (Beckman, Brea, CA, United States) as counts per minute.

### Statistical Analysis

Comparisons between multiple groups were performed using ordinary one-way ANOVA with the Holm-Sidak test for multiple comparisons. Comparisons between two groups were performed using an unpaired two-tailed *T*-test. *P-*values < 0.05 were considered significant. Statistical analysis was performed using GraphPad Prism software version 7.

## Results

### SIRT1 and Phosphorylated SIRT1 Expression Are Decreased During OLG Differentiation

**FIGURE 1:**
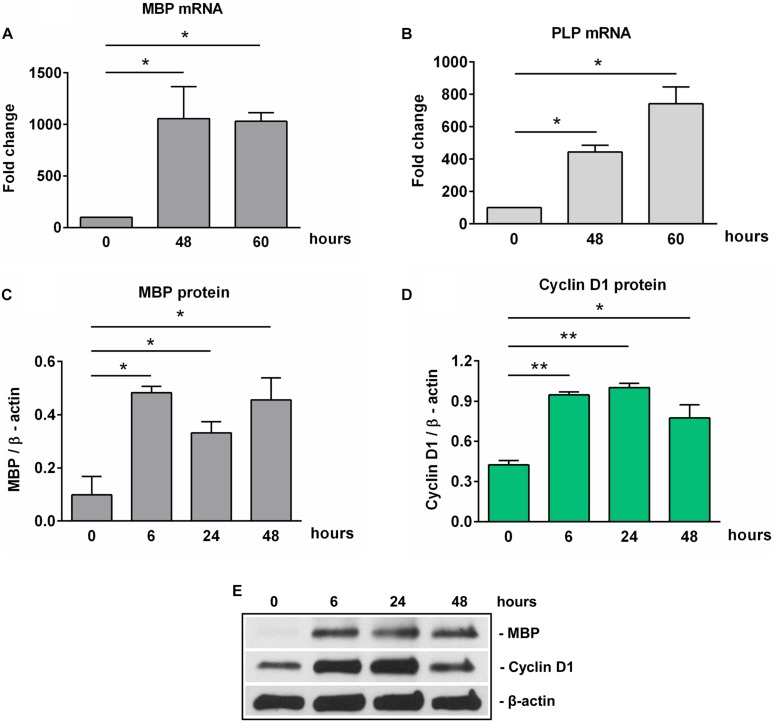
Expression of MBP, PLP, and cyclin D1 increases during OLG differentiation *in vitro*. OPCs purified from neonatal rat pups were cultured in an OLG differentiation medium, and expression of MBP and PLP mRNAs **(A,B)** was analyzed by real-time PCR. The expression of the mRNA at the beginning of the experiment (0 h) was considered to be 100, and the results are shown as -fold change. In addition, MBP **(C)** and cyclin D1 **(D)** protein expression was analyzed by Western blotting at the indicated time periods. The protein is expressed as a ratio to β-actin, and a representative Western blot is shown in **(E)**. Significantly higher levels of MBP and PLP associated with an increase of cyclin D1 in the first 24 h were found during OLG differentiation. The results are expressed as mean ± SEM (*n* = 3). *p < 0.05, **p < 0.01.

Oligodendrocyte precursor cells are highly proliferative cells actively involved in the generation of mature myelinating OLGs both during CNS development and in adults (24). Our real-time PCR and Western blot analyses revealed that OPCs differentiation toward OLGs *in vitro* was associated with a significant increase in the expression of MBP mRNA (Figure 1A) and protein (Figures 1C,E) as well as PLP mRNA (Figure 1B), in agreement with our previous findings (6). Both MBP and PLP are myelin components and markers of mature OLGs (25), and their expression indicates that the OPCs have successfully differentiated into OLGs *in vitro*.

Since the proliferation of OPCs was found to be associated with increased cyclin D1 levels (26, 27), we examined its expression in cultured OPCs and found a significant increase in protein level at 6 h (*p* = 0.005) and 24 h (*p* = 0.005), and then a trend toward decline at 48 h [although the levels remained significantly higher than those of OPCs at the start of the experiment (*p* = 0.01)] (Figures 1D,E), suggesting an initial entry of OPCs into the cell cycle, and then a tendency to shift toward cell cycle arrest as they assumed a more differentiated OLG phenotype (28).

**FIGURE 2:**
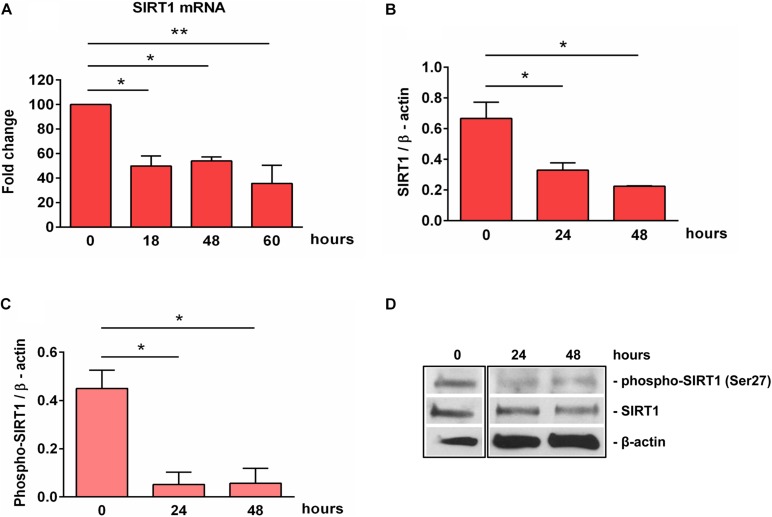
Expression of SIRT1 and phosho-SIRT1 is decreased during OLG differentiation. OPCs were cultured in differentiation medium and analyzed for the expression of SIRT1 mRNA **(A)**, SIRT1 protein **(B)**, or SIRT1 phosphorylated at serine 27 **(C)** at the indicated times. The expression of the mRNA at the beginning of the experiment (0 h) was considered to be 100, and the results are shown as -fold change **(A)**. The protein is expressed as a ratio to β-actin **(B,C)**, and a representative Western blot is shown in **(D)**. OLG differentiation is associated with decreased expression of SIRT1 and its active form phospho-SIRT1 (Ser27). Data are shown as mean ± SEM (*n* = 3). *p < 0.05; **p < 0.01.

We next examined the expression of SIRT1 during OLGs differentiation. High levels of SIRT1 mRNA and protein expression were found in OPCs. However, the expression of SIRT1 mRNA was significantly decreased at 18 h of OLGs differentiation (*p* = 0.01) when compared to OPCs levels at the start of the experiment (0 h), and it remained so up to 60 h (*p* = 0.003) (Figure 2A). We then asked whether similar changes occurred in SIRT1 protein expression. Our data showed that the initially high levels of SIRT1 protein seen in OPCs decreased significantly during differentiation at 24 h (*p* = 0.03) and 48 h (*p* = 0.03) (Figures 2B,D). We also measured the levels of p-SIRT1 at serine 27, a post-translational modification associated with increased SIRT1 protein stability and activation (29) and found that phospho-SIRT1 was significantly reduced after 24 h (*p* = 0.02) and remained significantly low at 48 h of differentiation (*p* = 0.02) (Figures 2C,D).

**FIGURE 3:**
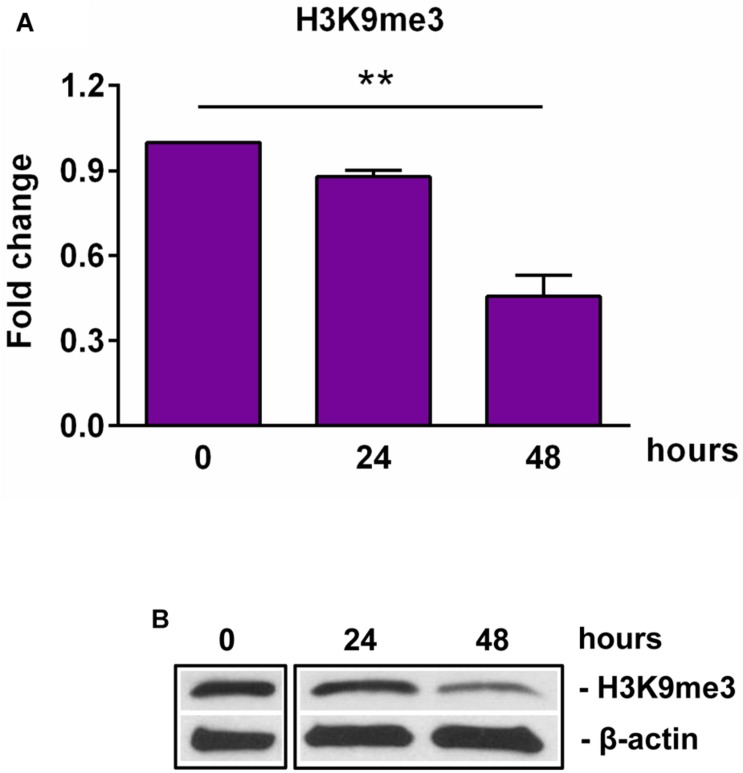
Expression of H3K9me3 during OLG differentiation. OPCs were cultured in differentiation medium and analyzed for the expression of H3K9me3 by Western blotting. The protein level was normalized to β-actin, and the results in **(A)** are shown as -fold change over 0 h, which was considered to be 1. A representative Western blot is shown in **(B)**. We found that OLG differentiation is associated with a decreased expression of H3K9me3. Data are expressed as mean ± SEM (*n* = 3). **p < 0.01.

SIRT1 levels are intricately coupled with the formation of a transcriptionally silent heterochromatin state. This is accomplished in part by the increased levels of histone 3 trimethylated at lysine 9 (H3K9me3) by the indirect action of SIRT1, which activates the histone methyltransferase SUV39H1, responsible for H3K9 trimethylation (30). Therefore, we looked at the levels of H3K9me3 during OLGs differentiation and found a significant decrease in its expression at 48 h (*p* = 0.007) (Figure 3), paralleling the decreasing SIRT1 and phospho-SIRT1 tendency.

Taken together, these findings suggest that OLGs differentiation is associated with an initial increase in the levels of cyclin D1 during the first 24 h and a decreased expression of SIRT1 and its active form phospho-SIRT1 (Ser27), which results in a decreased expression of the epigenetic marker H3K9me3, indicating a reorganization of the chromatin toward a global decrease of facultative heterochromatin.

### SIRT1 and Phospho-SIRT1 Levels Are Significantly Increased After Exposure to Sublytic C5b-9

**FIGURE 4:**
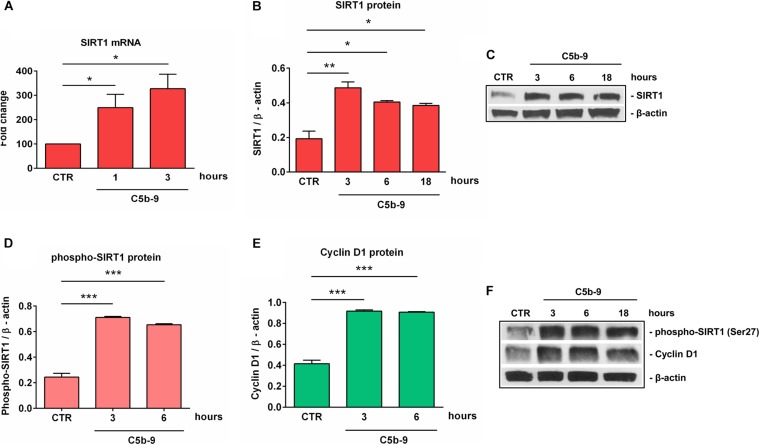
Induction of SIRT1, p-SIRT1 and cyclin D1 expression by sublytic C5b-9 in OLGs. OPCs were differentiated for 72 h, and OLGs were then exposed to sublytic levels of C5b-9 derived from either NHS or assembled purified complement components for the indicated periods of time. Expression of SIRT1 mRNA **(A)**, SIRT1 protein **(B)**, phospho-SIRT1 (Ser27) **(D)**, and cyclin D1 protein **(E)** was examined as described in section “Materials and Methods.” The expression of the mRNA at the beginning of the experiment (CTR) was considered to be 100, and the results are shown as -fold change **(A)**. The expression of SIRT1, phospho-SIRT1 and cyclin D1 proteins was normalized to β-actin, and representative blots are shown in **(C)** and **(F)**. We found that sublytic C5b-9 induced SIRT1 expression and activation, along with increased cyclin D1 expression. Results are expressed as mean ± SEM (*n* = 3). *p < 0.05; **p < 0.01; ***p < 0.001.

Given the alteration in SIRT1 protein expression that we observed during differentiation, we decided to concentrate our further efforts on investigating the effect of sublytic concentrations of C5b-9 on SIRT1 expression. We first asked whether sublytic C5b-9 had the ability to affect the protein expression of SIRT1. When we cultured differentiated OLGs in defined medium in the presence of C5b-9, we found that sublytic concentrations of C5b-9 induced a significant increase in SIRT1 mRNA expression when compared to CTR at 1 h (*p* = 0.04) and 3 h of stimulation (*p* = 0.02) (Figure 4A). We observed a similar increase in SIRT1 protein levels at 3 h (*p* = 0.005), 6 h (*p* = 0.01), and 18 h (*p* = 0.01) (Figures 4B,C). C5b6 had no effect on SIRT1 expression (data not shown). In addition, phospho-SIRT1 was also significantly increased after treatment with serum C5b-9 at 3 h (*p* = 0.0007) and 6 h (*p* = 0.0007) and remained upregulated up to 18 h after stimulation (Figures 4D,F).

**FIGURE 5:**
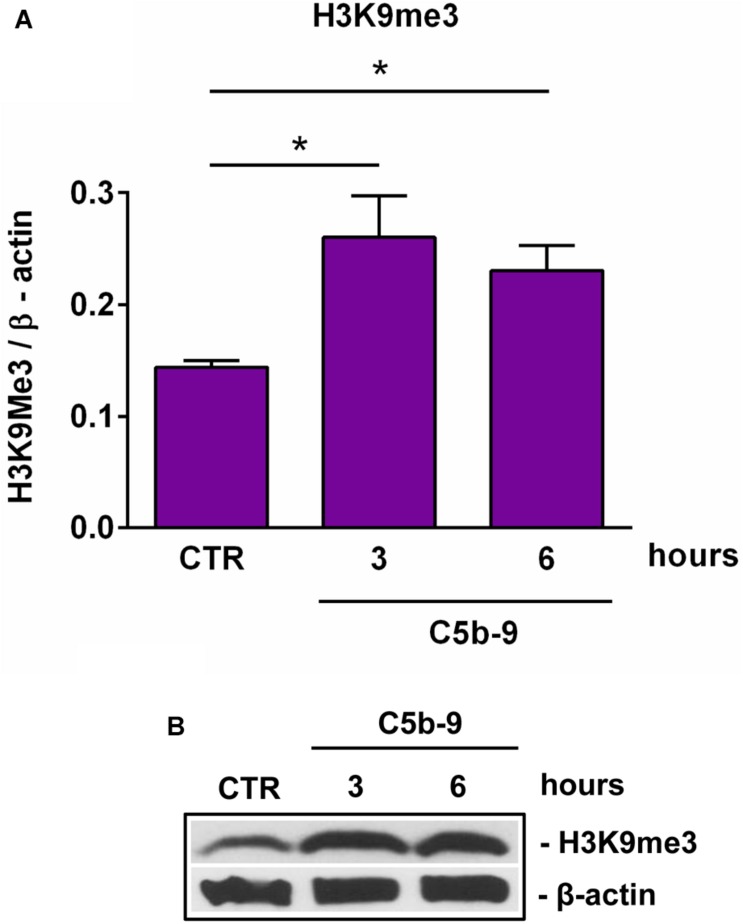
Effect of sublytic C5b-9 on H3K9me3 expression. OLGs were stimulated with sublytic levels of C5b-9 for the indicated periods of time and analyzed for the expression of H3K9me3 by Western blotting. The protein level is expressed as a ratio to β-actin **(A)**, and a representative blot is shown **(B)**. Our data show that H3K9me3 expression is significantly increased by C5b-9 stimulation. Data are shown as mean ± SEM (*n* = 3). *p < 0.05.

We have previously shown that sublytic C5b-9 stimulation of OLGs was associated with cell cycle activation and upregulation of cyclin-dependent kinases and proto-oncogenes (6). Therefore, we analyzed the expression of cyclin D1 and found that C5b-9 treatment led to a significant and sustained increase in its protein expression at 3 h (*p* = 0.0008) and 6 h (*p* = 0.0008) (Figures 4E,F). Given the increased levels of SIRT1, we also expected a similar trend for H3K9me3 and indeed, we found that H3K9me3 was significantly increased by C5b-9 stimulation at 3 h (*p* = 0.03) and 6 h (*p* = 0.04) (Figure 5).

### Effect of Sublytic C5b-9 on Myelin and OPCs Differentiation Markers

**FIGURE 6:**
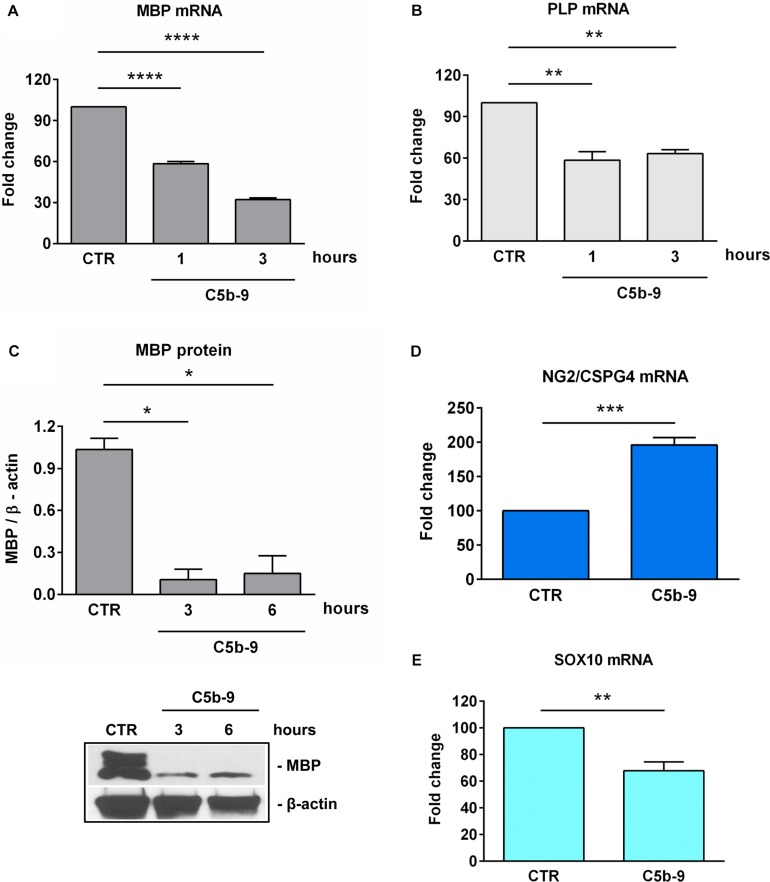
Effect of sublytic C5b-9 on progenitors and mature OLG markers. OLGs were stimulated with sublytic C5b-9 for the indicated periods of time and analyzed for the expression of MBP mRNA **(A)**, PLP mRNA **(B)**, and MBP protein **(C)**. OLGs were also stimulated with sublytic C5b-9 for 3 h and analyzed for the mRNA expression of NG2/CSPG4 **(D)** and Sox10 **(E)**. The expression of the mRNA at the beginning of the experiment (CTR) was considered to be 100, and the results are shown as -fold change **(A,B,D,E)**. MBP protein expression was normalized to β-actin, and a representative blot is shown in **(C)**. We observed a downregulation of the mature markers MBP, PLP, and SOX10 and a re-expression of NG2/CSPG4, suggesting the dedifferentiation of OLGs to an OPC-like phenotype after exposure to sublytic C5b-9. Data are expressed as mean ± SEM (*n* = 3). *p < 0.05; **p < 0.01; ***p < 0.001; ****p < 0.0001.

On the other hand, as expected (6), treatment with sublytic C5b-9 was associated with a significant decrease in the expression of MBP mRNA (Figure 6A) and protein (Figure 6C) as well as PLP mRNA (Figure 6B). We have also analyzed the expression of NG2, which is also known as CSPG4 and is highly expressed in OPCs (31). NG2/CSPG4 expression was significantly increased after 3 h of C5b-9 treatment (*p* = 0.0008) (Figure 6D). On the other hand, the expression of Sox10, a transcriptional factor crucial for OLGs terminal differentiation and expression of myelination genes (32, 33) was significantly decreased after 3 h of C5b-9 treatment (*p* = 0.003) (Figure 6E).

These findings suggest that sublytic C5b-9 induced SIRT1 expression, along with a state of dedifferentiation in the OLGs featuring an OPC-like phenotype defined by the downregulation of the mature markers MBP, PLP and SOX10 and a re-expression of the NG2/CSPG4, as well as cell cycle re-entry and an increase in global heterochromatin formation.

### C5b-9-Induced SIRT1 Expression Is c- jun-, PKC-, and PI3K / Akt-Dependent

**FIGURE 7:**
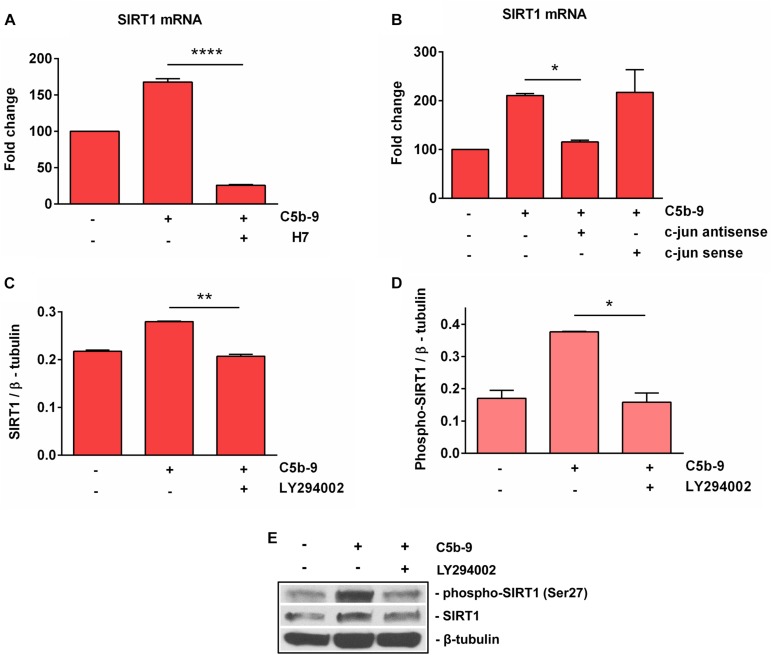
Effect of c-jun, PKC, and PI3K inhibition on SIRT1 expression. OLGs were exposed to sublytic levels of C5b-9 either alone or after preincubation with a protein kinase C (PKC) inhibitor (60 μM H7, preincubated for 30 min) **(A)**, a c-jun antisense oligonucleotide (20 μM, preincubated for 4 h) **(B)**, or LY294002 (10 μM, preincubated for 1 h) to inhibit the PI3K/Akt pathway **(C,D)**. OLGs were then analyzed for the expression of SIRT1 mRNA by real-time PCR and for SIRT1 protein and phospho-SIRT1 (Ser27) by Western blotting. The expression of the mRNA at the beginning of the experiment (untreated cells) was considered to be 100, and the results are shown as -fold change **(A,B)**. The protein levels are expressed as ratios to β-tubulin **(C,D)**, and a representative blot is shown in **(E)**. The results show that sublytic C5b-9-induced expression of SIRT1 is dependent on PKC, PI3K-Akt signaling pathways, and c-jun activity. Data are shown as mean ± SEM (*n* = 3). *p < 0.05; **p < 0.01; ****p < 0.0001.

It was shown that sublytic C5b-9 stimulation of OLGs activates a plethora of intracellular signaling pathways involved in cell cycle activation, including PI3K / Akt (11), and PKC (7, 8). In order to further investigate the mechanisms of C5b-9-induced SIRT1 expression in OLGs, we used specific inhibitors to examine the signaling pathways that might be involved in its expression. The C5b-9-induced expression of SIRT1 mRNA was inhibited by H7, a PKC inhibitor (*p* < 0.0001) (Figure 7A). In addition, c-jun antisense treatment led to a significant reduction (*p* = 0.04) in sublytic C5b-9-induced expression of SIRT1 mRNA (Figure 7B), whereas c-jun sense oligonucleotide had no effect. PI3K inhibition by the potent inhibitor LY294002 also significantly reduced the expression of both SIRT1 protein (Figures 7C,E) (*p* = 0.002) and phospho-SIRT1 (*p* = 0.02) (Figures 7D,E). On the other hand, pertussis toxin (PTX) treatment, which inhibits G_*i*_ protein signaling, had no effect on SIRT1 mRNA expression (data now shown). These data indicate that the sublytic C5b-9-induced expression of SIRT1 is dependent on PKC, PI3K-Akt signaling pathways, and c-jun activity.

### C5b-9-Induced Cell Cycle Activation in OLG Is SIRT1-Dependent

**FIGURE 8:**
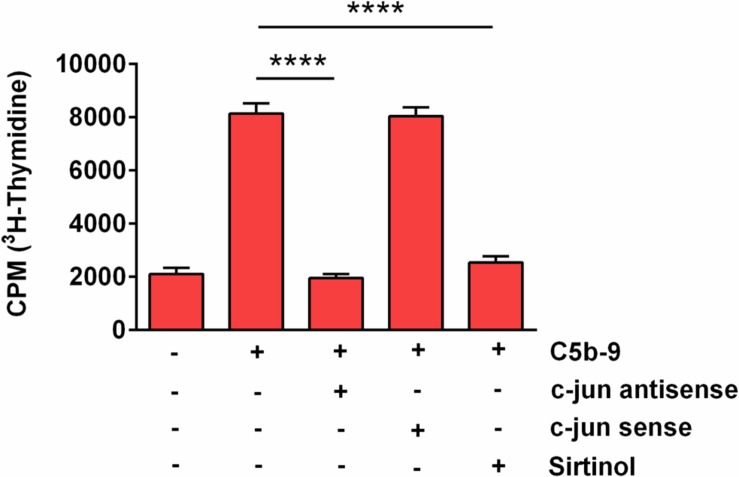
Effect of SIRT1 and c-jun inhibition on C5b-9-induced [^3^H] thymidine incorporation. OLGs were incubated with C5b-9 assembled from purified complement components, either alone or after a 4-h preincubation with a c-jun antisense oligonucleotide (20 μM), a c-jun sense oligonucleotide (20 μM), or sirtinol (200 μM), a SIRT1 inhibitor. The cells were then incubated with [^3^H] thymidine and processed, and radioactivity was detected by liquid scintillation as counts per minute (CPM). Our data show that S-phase entry induced by sublytic C5b-9 in OLGs is c-jun and SIRT1-dependent. Data are expressed as mean ± SEM (*n* = 3). ****p < 0.0001.

Since our results showed that SIRT1 upregulation in response to sublytic C5b-9 is also c-jun-dependent, we asked whether SIRT1 is also involved in S-phase induction. We therefore performed a [^3^H] thymidine incorporation assay to probe for DNA synthesis in OLGs after they were stimulated with C5b-9, alone or after pretreatment with sirtinol, a potent SIRT1 inhibitor. Pretreatment with sirtinol abolished C5b-9-induced DNA synthesis (*p* < 0.0001) (Figure 8). In addition, c-jun antisense significantly decreased the C5b-9-induced thymidine incorporation (*p* < 0.0001) (Figure 8). In contrast, c-jun sense had no effect. These data suggest that S-phase entry induced by sublytic C5b-9 in OLGs is c-jun and SIRT1-dependent.

## Discussion

To our knowledge, our study is the first to show that SIRT1 is induced in OLGs exposed to sublytic C5b-9 *in vitro* and that it regulates cell cycle activation and possibly dedifferentiation of the OLGs toward an OPC-like phenotype. We found that sublytic C5b-9 stimulation of differentiated OLGs, which are normally in the G0/G1 state, both activated the cell cycle and induced cyclin D1, SIRT1, and H3K9me3 expressions to levels similar to those seen in OPCs. In addition, formation of sublytic C5b-9 induced a marked reduction in mRNA encoding MBP and PLP, which are major structural proteins of CNS myelin. This dedifferentiation of OLGs seen in vitro was reversible after the removal of the C5b-9 from the culture medium. We have shown previously that C5b-9 induces a rapid degradation of MBP and PLP mRNA, indicating that post-transcriptional regulation plays an important role in modulating their expression (7). When sublytic C5b-9 was removed from the medium, the expression of MPB and PLP was enhanced by the trophic factors present in the differentiation medium (7).

DNA synthesis was also abolished by sirtinol, a SIRT1 inhibitor, indicating that C5b-9-induced cell cycle activation in OLGs is SIRT1-dependent. SIRT1 can both induce and repress the cell cycle as well as cyclin D1 expression in a cell type- and context-dependent manner (34–36). In the present study, we found a peak of cyclin D1 expression at 24 h, suggesting that OPCs were initially in a highly proliferative state. Cyclin D1 is a marker of cell cycle entry and a key regulator of the G1-S transition, and its increased expression was associated with OPCs proliferation, both *in vitro* as well as *in vivo.* Interestingly, cyclin D1 is one of the early genes activated in OPCs treated with neuroblastoma-conditioned medium (37), and its expression is increased under conditions associated with high OPCs proliferation rates, such as in oligodendroglial tumors (38). Others have found that its expression is induced by growth factor-mediated activation of several intracellular signaling pathways, including PI3K/Akt and extracellular-regulated kinase 1/2 (ERK1/2) (39). On the other hand, its expression declines as OLGs progress toward terminal differentiation (28). More studies are needed to determine whether SIRT1 is involved in regulating cyclin D1 expression in OLGs through transcriptional activation or is modulating its levels through deacetylation.

Several studies have recently shown that SIRT1 regulates the proliferation of OPCs (18–20). Xiang et al. have shown that overexpression of SIRT1 in cultured mouse OPCs promotes their differentiation toward OLGs through the upregulation of peroxisome-proliferator-activated receptor gamma co-activator 1 alpha (PGC1α), and this process is inhibited in the presence of mutant huntingtin (40). On the other hand, in a chronic neonatal hypoxia mouse model, Jablonska et al. have shown that SIRT1 promotes OPC proliferation through the deacetylation of the retinoblastoma (Rb) protein and Cdk2, thereby increasing Cdk2’s activity. The Cdk2/cyclin E complex hyper-phosphorylates Rb, resulting in dissociation of the E2F1 transcription factor and S-phase entry. Inhibition of SIRT1 in the chronic neonatal hypoxia mouse model results in OLG differentiation, suggesting that SIRT1 may be important in maintaining an endogenous pool of proliferating OPCs in the developing white matter (19). In another study, Rafalski et al. have reported that conditional deletion of SIRT1 in adult neural stem cells increases the generation of new OPCs and fully myelinating OLGs, ameliorating both remyelination and clinical status in a mouse model of lysolecithin-induced demyelination (18). In addition, SIRT1 inactivation in the CNS enhances the rate of remyelination in demyelinating injury and minimizes the severity of clinical symptoms in chronic experimental allergic encephalomyelitis (EAE) (18). A recent study in EAE mice has also found that SIRT1 is co-localized with NG2^+^ and platelet-derived growth factor receptor alpha (PDGFRα)^+^ OPCs in cerebellar white matter in close proximity to demyelinating areas during the chronic stage of the disease, and SIRT1 inhibition results in an enhanced proliferation of OPCs without affecting their ability to differentiate (20). These findings suggest that OPC differentiation occurs in the absence of SIRT1 and that the role of SIRT1 in OPC proliferation may be region- and disease-specific. Nevertheless, the abovementioned results in EAE mice are in contrast with data published by Nimmagadda et al. (41), which showed that overexpression of SIRT1 in neurons attenuates EAE clinical symptoms and reduces inflammatory cell infiltration, demyelination, and axonal injury. In addition, treatment of EAE with resveratrol, a naturally occurring polyphenol that activates SIRT1, reduces disease severity (42, 43). Taken together, these studies suggest that inactivation of SIRT1 may promote OLG differentiation and thus may be a target for improving symptoms of demyelinating diseases; on the other hand, increased SIRT1 expression in other brain cells during EAE may be anti-inflammatory and neuroprotective.

**FIGURE 9:**
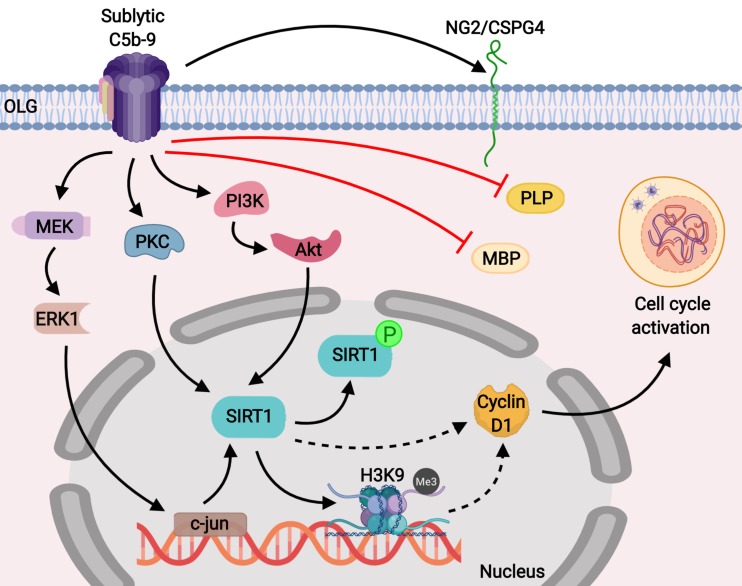
Schematic representation of the role of SIRT1 in C5b-9-mediated cell cycle activation in OLGs. Our results show that sublytic C5b-9 induces the expression of NG2/CSPG4, a marker for OPCs, and mediates cell cycle activation by inducing SIRT1 through the c-jun, protein kinase C, and PI3K/Akt signaling pathways. In addition, sublytic C5b-9 was found to significantly down-regulate the expression of MBP and PLP. SIRT1 is known to deacetylate lysine 9 of histone H3 and indirectly promotes its trimethylation (H3K9me3), resulting in a facultative heterochromatin state. We speculate that this result leads to the transcriptional inhibition of cell cycle inhibitors, allowing for the expression of cyclin D1 and promoting cell cycle activation. Sublytic C5b-9 also activates proto-oncogenes, leading to transcriptional and cell cycle activation. Inhibition of c-jun with antisense c-jun leads to inhibition of sublytic C5b-9-induced SIRT1 expression and cell cycle activation.

We found that the decreased expression of SIRT1 during OLG differentiation was associated with a decreased level of H3K9me3, which serves as a marker of facultative heterochromatin, a compact and closed status of chromatin that is associated with transcriptional repression (44). It has been shown that SIRT1 promotes the trimethylation of lysine 9 on H3 (H3K9) indirectly by two main mechanisms: by deacetylating H3K9 and by directly enhancing the activity and stability of the histone methyltransferase SUV39H1, the enzyme responsible for the trimethylation of H3K9 (30, 45). It is well known that epigenetic modifications play a significant role in OLG differentiation, and SIRT1, together with other HDACs such as HDAC1 and HDAC2, is involved in controlling the status of chromatin and regulating transcription during OPC proliferation and differentiation (46, 47). Interestingly, it has been shown that HDACs can coordinately regulate OPC proliferation and differentiation toward myelinating OLGs at different stages, in cooperation with various transcriptional activators or repressors (47). For example, HDACs can be recruited to repressor complexes that inhibit myelin gene transcription in OPCs but later promote such expression in myelinating OLGs by inhibiting transcriptional repressors (46, 48). On the other hand, a number of studies using various HDAC inhibitors have shown that HDAC blockade can either prevent or promote OLG differentiation and myelination in a time- and context-dependent manner (47, 48). It is possible that the facultative heterochromatin state associated with high H3K9me3 expression after C5b-9 stimulation observed in our study contributes to the transcriptional inhibition of myelin-specific genes such as MBP and PLP and of cell cycle inhibitors, resulting in increased cyclin D1 expression and cell cycle progression (Figure 9).

It has also been reported that sublytic C5b-9 is able to activate a cascade of signaling pathways in OLGs, including those involving PI3K/Akt, PKC, and two mitogen-activated protein kinase (MAPK) family members, ERK1 and c-jun N terminal kinase 1 (JNK1) (7, 9, 11). We have also found that C5b-9-induced SIRT1 expression in OLGs depends on the activation of several signaling pathways that rely on kinases such as PI3K/Akt and PKC. PKC has a complex role in OLG differentiation, since it has been shown to prevent differentiation when its expression is induced in OPCs (49). Studies have also found that PKC is involved in lead-, ethanol- and myelin debris-induced inhibition of OLG differentiation (50–52). Nevertheless, others have found that PKC is active and phosphorylates several downstream targets associated with OLG differentiation (53, 54). The PI3K/Akt signaling pathway is also important for OLG differentiation (55) and for sublytic C5b-9-induced survival of OLGs [10] [11]. Others have shown that SIRT1 deacetylates Akt to promote binding to phosphatidylinositol 3,4,5 triphosphate in the plasma membrane and Akt activation (56). We speculate that sublytic C5b-9 induction of Akt in OLGs is dependent on SIRT1-induced deacetylation of Akt, and this deacetylation contributes to cell cycle activation. Sublytic C5b-9 activate proto-oncogenes, including c-jun (6, 57, 58) leading to transcriptional and cell cycle activation. In our study, inhibition of c-jun with an antisense oligonucleotide led to the inhibition of sublytic C5b-9-induced SIRT1 expression and suppression of cell cycle activation, indicating that c-jun is needed for SIRT1 expression. It is unclear whether c-jun is required for transcriptional activation of SIRT1, since we are unaware of any published data to sustain such a role for c-jun. Thus, more work needs to be done to clarify the role of c-jun in SIRT1 activation.

## Conclusion

In conclusion, we have found that sublytic C5b-9 induces cell cycle activation in OLGs as well as a dedifferentiated state by inducing the expression of SIRT1 through multiple protein kinase signaling pathways. This immature phenotype that resembles OLG precursors may prove helpful in facilitating OLG survival in an inflammatory environment and providing an opportunity for the cells to regain their remyelination capacity once the inflammation subsides. However, more studies are needed to elucidate the intrinsic molecular mechanisms of SIRT1-mediated OLG survival and to better understand the role of SIRT1 in the remyelination of surviving OLGs in chronic MS lesions.

## Data Availability Statement

The datasets generated for this study are available on request to the corresponding author.

## Ethics Statement

The animal study was reviewed and approved by the Institutional Animal Care and Use Committee-University of Maryland School of Medicine, Baltimore, MD, United States.

## Author Contributions

HR and VR designed the study. AT, GR, DB, SV, AB, and FA performed the experiments. AT, VR, and HR wrote the manuscript. All authors approved the manuscript.

## Conflict of Interest

The authors declare that the research was conducted in the absence of any commercial or financial relationships that could be construed as a potential conflict of interest.

## Abbreviations

CNS: central nervous system
CSPG4: chondroitin sulfate proteoglycan 4
EAE: experimental autoimmune encephalomyelitis
ERK: extracellular-regulated kinase
MBP: myelin basic protein
MS: multiple sclerosis
NG2: neuron glial antigen 2
OLG: oligodendrocyte
OPC: oligodendrocyte precursor cell
PI3K: phosphoinositide 3-kinase
PKC: protein kinase C
PLP: proteolipid protein
p-SIRT 1: phosphorylated SIRT1
SIRT1: sirtuin 1.
